# DNA Methylation and Type 2 Diabetes: the Use of Mendelian Randomization to Assess Causality

**DOI:** 10.1007/s40142-019-00176-5

**Published:** 2019-11-15

**Authors:** Diana L. Juvinao-Quintero, Marie-France Hivert, Gemma C. Sharp, Caroline L. Relton, Hannah R. Elliott

**Affiliations:** 1 MRC Integrative Epidemiology Unit at the University of Bristol, Oakfield House, Oakfield Grove, Bristol BS8 2BN, UK; 2 Population Health Sciences, Bristol Medical School, University of Bristol, Oakfield House, Oakfield Grove, Bristol BS8 2BN, UK; 3 Division of Chronic Disease Research Across the Lifecourse, Department of Population Medicine, Harvard Medical School and Harvard Pilgrim Health Care, Boston, USA; 4 Bristol NIHR Biomedical Research Centre, Bristol, UK

**Keywords:** Type 2 diabetes, DNA methylation, Biomarkers, Causal inference methods, Mendelian randomisation

## Abstract

**Purpose of Review:**

This review summarises recent advances in the field of epigenetics in order to understand the aetiology of type 2 diabetes (T2D).

**Recent Findings:**

DNA methylation at a number of loci has been shown to be robustly associated with T2D, including *TXNIP, ABCG1, CPT1A*, and *SREBF1*. However, due to the cross-sectional nature of many epidemiological studies and predominant analysis in samples derived from blood rather than disease relevant tissues, inferring causality is difficult. We therefore outline the use of Mendelian randomisation (MR) as one method able to assess causality in epigenetic studies of T2D.

**Summary:**

Epidemiological studies have been fruitful in identifying epigenetic markers of T2D. Triangulation of evidence including utilisation of MR is essential to delineate causal from non-causal biomarkers of disease. Understanding the causality of epigenetic markers in T2D more fully will aid prioritisation of CpG sites as early biomarkers to detect disease or in drug development to target epigenetic mechanisms in order to treat patients.

## Introduction

Type 2 diabetes (T2D) is a metabolic disorder characterised by hyperglycemia due to β cell dysfunction and insulin resistance [1, 2]. T2D affects 8.3% of the adult population worldwide and is one of the most common non-communicable diseases of current times [3, 4]. Aetiologically, T2D arises in response to a combination of genetic predisposition and environmental or lifestyle factors. The genetic origins of T2D have long been supported by family and twin studies [5]. The most recent GWAS meta-analysis in T2D identified > 400 genetic risk variants explaining 15–18% of the heritability of the disease [6, 7]. Most of the T2D-risk variants identified to date act through effects on β cell function [5, 8] and to a lesser extent through effects on insulin resistance and obesity [1].

Epigenetic modifications include DNA methylation (DNAm), post-translational modification of histone proteins, and non-coding RNAs (ncRNAs) [9–11]. More rarely studied modifications include 5-hydroxymethylcytosine (5hmC), 5-formylcytosine (5fC), and 5-carboxylcytosine (5caC) which are sequentially produced by oxidation during enzymatic demethylation [12]. Epigenetic modifications act at the interface between the environment and coordinated transcriptional control and may also contribute to T2D disease risk. This may be partly via genetic influences on epigenetic modifications, but epigenetic response to the influence of environmental and lifestyle exposures is likely to be predominant. Evidence supporting this comes from greater epigenetic variation observed in population studies compared with that in discordant monozygotic twins [13].

The most common epigenetic modification analysed in epidemiological studies of complex diseases is DNAm [10, 11, 14], mainly found at CpG dinucleotides [9–11]. Growing evidence supports an association between T2D and DNA methylation variation measured before [15] and after disease onset [16•]. However, it is unknown whether epigenetic markers (particularly those identified in non-target tissues) play a causal role in the development of T2D, if they are a consequence of disease status, or are due to residual confounding [9]. Moreover, the epigenetic signature observed in collected samples that include mixed cell types can influence associations that may or may not be completely considered in all observational studies despite currently available biostatistical tools [17–20].

This review presents an overview of current evidence around T2D and epigenetics, with a primary focus on DNA methylation and epidemiological studies in humans. It describes inherent limitations of epigenetic studies in ascertaining causality and provides examples of studies that have attempted to address these limitations by implementing causal inference methods such as Mendelian randomisation.

## General Considerations in the Design of DNA Methylation Studies

The use of DNAm in epidemiological studies imposes some constraints, particularly when the aim is to identify evidence for a causal role of DNAm on disease. Common challenges include tissue specificity, confounding, effect modification, and statistical power to identify methylation variable sites with enough interindividual variation to be informative between comparison groups [21, 22]. Additionally, technological advances have moved the field of epigenetics from the study of a few CpG sites within specific genes, towards the genomewide assessment of variation in DNAm at single-base resolution [23•]. Genome-wide studies of DNAm allow ascertainment of disease-relevant variation more comprehensively than candidate gene studies. However, they also increase the multiple testing burden (~ 400–800 K sites analysed), requiring larger samples to robustly identify small effect sizes [10, 24]. In addition, current array-based methods for genome-wide assessment only cover < 2% of the total methylation sites available in the genome [11]. Prediction of DNAm at unmeasured sites is more difficult to achieve than for common genetic variants (SNPs) measured using arrays due to the more complex correlation structure of DNAm over specific genomic regions and CpG site densities, and due to the temporal and tissue-specific variation of DNAm [25].

In principle, when the aim is to study aetiology, DNAm markers should be studied in disease-relevant tissues (i.e. insulin-responsive tissues or insulin-producing β cells) to validate their role in the causal pathway to disease [26]. For T2D, such tissues might include pancreatic islets, liver, adipose tissue, or skeletal muscle. However, internal tissues are more difficult to access than peripheral blood, especially at scale [23•, 27•]. In addition, tissue contamination due to ongoing inflammatory processes in obese patients with T2D (i.e. infiltration of blood cells into adipose tissue) or due to sample derivation should be considered. Cellular heterogeneity can influence the comparison of DNAm across tissues. Currently, cell-type deconvolution methods are being developed to estimate the proportion of cells in tissues different from peripheral blood [28]. These methods could aid in discriminating the inflammatory proportion in internal target tissues. Conversely, when the aim is primarily for prediction (irrespective of mechanistic role), it may be appropriate to study more accessible tissues like blood, saliva, buccal cells, cells in urine, skin cells, and faeces, which are commonly collected in large-scale epidemiological studies [11, 14]. Replication of signals detected in accessible tissues within internal target tissues may provide further evidence of their biological role in T2D pathophysiology and their potential use in diagnostics or therapeutics.

Methylation may be influenced by environmental factors related to the disease being studied, by the disease itself or disease treatment, and this possibility of reverse causation means that it can be difficult to discern causality in cross-sectional studies [11, 14, 22]. Studies that measure DNAm before clinical detection of T2D can be valuable in this regard [14, 23•, 26], as they reduce the likelihood of confounding by reverse causation (i.e. disease influencing DNAm variation). However, longitudinal studies are expensive and uncommon when compared with cross-sectional studies [11, 14, 23•] and do not completely eliminate risk of reverse causation due to subclinical manifestations of the disease [10]. In T2D, several studies have replicated markers of predisposition detected longitudinally, in cross-sectional case-control studies [27•, 29•]. This suggests that variation in methylation detected prior to disease onset is not necessarily indicative of causality since the observed associations can still be influenced by unmeasured environmental or genetic confounders [10]. Alternatively, the analysis of glycemic and other T2D-related traits has been useful to identify markers of predisposition and potential prediction of T2D in disease-free participants [15, 30–32].

Another challenge that can hamper the identification of epigenetic mechanisms in T2D is sample size. Adequate sample sizes required to detect associations in epigenetic epidemiology studies have been estimated to be in the region of ~ 1000 samples, whereas the vast majority of published literature in this field falls well below this threshold [24]. Studies of the epigenetic epidemiology of T2D have tended to be smallscale (< 100 participants) and therefore suffer from low statistical power. More recently, replication and meta-analysis of associations across studies have become more common [11, 22]. Replication and meta-analysis are facilitated by the emergence of large consortia of cohorts that use similar profiling methods for DNAm and standardised protocols for data preprocessing and analysis [10, 11, 22].

## Summary of Current Knowledge from Human DNA Methylation Studies in T2D

### Candidate Gene Analyses

Epigenetic studies based on candidate loci rely on previous knowledge in order to select the genomic region(s) to study [33]. Several candidate gene studies have been conducted to date that use T2D relevant tissues such as human pancreatic islets [34–37] or skeletal muscle biopsies [38] from T2D donors and appropriately selected controls. These studies have identified differential methylation at genes related to insulin activity (*INS* and *GLP1R*) [35, 37], β cell function (*PDX1*) [36], and energy balance (*PPARGC1A*) [34]. In addition, there have been several candidate gene studies aimed at identifying methylation variable loci using peripheral blood DNA [39–43] (Table 1), and they have been reviewed in detail elsewhere [23•, 33, 44]. One recent example is the study conducted by Seman et al. [45] looking at differential methylation at the promoter of *SLC30A8*, a pancreas-specific zinc efflux transporter [23•]. The authors identified hypermethylation of five CpG sites in *SLC30A8* in T2D cases (*n* = 509) versus controls (*n* = 441) in a large Malay study [45].

Overall, results of the candidate gene approach have shown that differential methylation at the promoter regions of well-established genetic loci for T2D is associated with T2D risk. Two hypotheses arise from this observation (i) that genetic or epigenetic perturbation at the locus of interest may both contribute to disease risk (an additive effect), or (ii) that DNAm might be important in mediating the effect of known genetic variants and T2D (a mediating effect). However, recent genetic studies of DNAm using peripheral blood have provided less evidence that DNAm is mediating the effect between known T2D-SNPs and T2D risk [46, 47•], with the exception of methylation at the T2D candidate loci *KCNJ11, WFS1* [47•], and *KCNQ1* [46].

### Epigenome-Wide Approaches

Epigenome-wide association studies (EWAS) of T2D have been conducted to identify novel markers of disease incidence or prevalence (Table 1) using longitudinal and cross-sectional studies, respectively.

#### Blood DNA Methylation as a Marker of Incident T2D

One of the earliest genome-wide studies of T2D was conducted by Toperoff et al. using a microarray-based technology [48]. This study identified several differentially methylated regions (DMRs) associated with T2D that were enriched in genetic loci previously reported for T2D. In a second prospective cohort, the authors identified that hypomethylation of one of the associated regions (in *FTO*) was observed in young individuals who later progressed to T2D, relative to the individuals who stayed healthy [48].

More recently, Chambers et al. conducted the largest study to date looking at differential DNAm in association with future T2D. This multiethnic longitudinal study included samples of Indian Asian (discovery) and European (replication) origin [16•]. Hypomethylation at CpG sites in *TXNIP* (cg19693031), *PHOSPHO1* (cg02650017), and *SOCS3* (cg18181703), and hypermethylation at *SREBF1* (cg11024682) and *ABCG1* (cg06500161) were associated with greater risk of developing T2D over the ~ 8.5-year follow-up [16•] (Table 1). In addition, these five CpG sites were combined into a methylation risk score that predicted a 3.51 (95% CI = 2.79–4.42) increased risk of future T2D among Indian Asians. This association was independent of adiposity and the homeostasis model assessment for insulin resistance (HOMA-IR) [16•]. Due to the short time elapsed between sample recruitment and disease detection (mean = 8.5 years), it is possible that some individuals with subclinical disease could have been misclassified as disease-free at baseline in this study. However, an independent study by Dayeh et al. [54] replicated associations at *ABCG1* and *PHOSPHO1* using samples from the Botnia prospective family-based study. In this study, unaffected participants were on average followed-up during 8.1 years until clinical detection of T2D. Dayeh et al. also demonstrated that methylation of ABCG1 and *PHOSPHO1* was associated with other metabolic risk factors [54]. To further support a mechanistic role of methylation at *ABCG1* and *PHOSPHO1* on T2D, Dayeh et al. compared the association at these sites using target tissues for T2D, identifying consistency in the direction of effect between blood and adipose tissue for ABCG1, and between blood and skeletal muscle for *PHOSPHO1* [54]. Lastly, gene expression of *ABCG1* was inversely correlated with methylation of *ABCG1* in muscle but not in peripheral blood, whilst no correlation between these traits was identified for *PHOSPHO1* in any of the tissues interrogated [54].

#### Blood DNA Methylation as a Marker of Prevalent T2D

The vast majority of T2D EWAS have used a cross-sectional case-control design to compare DNAm in diagnosed T2D cases and controls who are presumed to be disease-free [15, 27•, 29•, 48, 49, 52, 55, 56]. Some of the CpG sites identified in studies of prevalent T2D (*TXNIP, ABCG1*, and *SREBF1*) have also been reported in studies of incident T2D [16•]. This might be explained by misclassification of subclinical T2D as disease-free (as discussed above), or it might also reflect a causal effect of DNAm at these sites on T2D, which persists once the disease is established. It could also reflect persistent confounding factors, including underlying genetic effects on T2D and DNAm.

In EWAS of T2D using peripheral blood, the CpG site that has most commonly been associated with T2D, independently of body mass index (BMI), is *TXNIP* (cg19693031) [15, 27•, 29•, 55, 56•]. For example, in a case-control study of ~ 1500 adults, Florath et al. [29•] identified 39 T2D-associated CpG sites in a discovery cohort, with replication of methylation differences in a second subset of the cohort for a signal mapping to *TXNIP*. At this site, T2D cases are consistently hypomethylated compared with controls according to studies in Europeans [16•, 29•, 55], Indian Asians [16•], Mexican Americans [15], Arabs [49], and Ghanaians [56•]. The generalisability of the association at *TXNIP* across populations supports the potential clinical use of this site as a biomarker of T2D risk. In addition, methylation of *TXNIP* appears to be inversely associated with HbA1c [29•, 55, 56•] and fasting glucose [29•], leading to the hypothesis that sustained hyper-glycemia may be one of the factors driving hypomethylation of *TXNIP* [57•]. *TXNIP* is the *thioredoxin interacting protein*, which is responsive to glucose concentrations in the cell. The protein is overexpressed in humans and animals with T2D [23•], and its function has been linked to vascular complications by modulating angiogenesis and inhibiting the vascular endothelial growth factor (VEGF) [23•].

In addition to *TXNIP*, reproducible CpG sites in T2D have been reported at *ABCG1* (cg06500161), *C7orf50* (cg04816311), and CPT1A (cg00574958) [56•], and more population-specific sites have been discovered at *DQX1* (cg06721411), *TPM4* (cg07988171), and *MSI2* (cg23586172) in samples of Qatari [49], Ghanaian [56•], and Korean origin [58], respectively.

Considering the growing evidence of DNAm as a marker of T2D predisposition and state, Walaszczyk and colleagues evaluated the replicability of the CpG sites most recently reported in the literature in association with T2D, HbA1c, and fasting glucose [27•]. Associations considered for replication were CpG sites that had been previously reported across ethnic groups and tissues [27•]. The target sample for replication was a case-control subsample (*n* = 200, cases = 100, controls = 100) of the LIFELINES prospective population-based study from the Netherlands with availability of whole blood DNAm [27•]. Replication was achieved for T2D-associated CpG sites in *ABCG1, LOXL2, TXNIP, SLC1A5*, and *SREBF1*, and for fasting glucose-associated CpG sites in *ABCG1* and *CCDC57* (Table 1). Additionally, the authors reported poor cross-tissue consistency in T2D-associated CpG sites, as none of the associations previously reported in the liver, pancreas, and adipose tissue were replicated in blood.

EWAS of prevalent and incident T2D using peripheral blood DNA have also demonstrated that methylation variable loci in T2D do not overlap with previous GWAS loci for the disease. Thus, DNAm may influence biological mechanisms of tissue response to hyperglycemia different from those implicated by genetic studies, which appear to be primarily associated with β cell function and insulin activity.

#### EWAS of T2D in Disease-Relevant Tissues

EWAS of T2D have also been conducted in disease-relevant tissues and have recently been reviewed by Davegårdh et al. [57•]. Sample sizes used in these studies tend to be smaller due to tissue or cell availability. However, replicated associations between DNAm and T2D, or T2D-related traits (i.e. obesity, BMI, fat distribution, diet, exercise), have been identified in adipose tissue [50, 51, 59–64], islets [53, 65–67], skeletal muscle [59, 68, 69], and liver tissue [70, 71] (Table 1). However, there has been little overlap between CpG sites identified in EWAS of these tissues and EWAS of blood, except for at *ABCG1*, which was hypermethylated in blood and in adipose tissue of T2D cases [54], and *MSI2*, which was hypomethylated in blood and in pancreatic islets of T2D donors [58]. Conversely, unlike in EWAS of T2D in blood, some of the methylation loci identified in EWAS of disease-relevant tissues overlap with GWAS loci for T2D [60–62, 67].

### The genetics of epigenetics and using Mendelian randomisation as a method to infer the causal role of methylation variation in T2D

The genetics of epigenetics and using Mendelian randomisation as a method to infer the causal role of methylation variation in T2DDisease-associated methylation variation may be causal or consequential [10, 72]. Several mechanisms explain how variation in methylation arises prior to disease onset, for example via stochastic changes during development, or in response to environmental exposures at any stage of the life course [12]. However, variation in methylation detected prior to disease onset is not always an indicator of causality [12]. Because observational studies do not allow us to distinguish between causal and consequential epigenetic variation, following robust replication of findings, triangulation of methods for assessing causality is increasingly informative [73, 74]. Methods include parental negative control studies [75], cross-cohort comparisons [73], matched within sibship designs [76], and Mendelian randomisation (MR) [9, 11, 74, 77–79]. MR is increasingly widely applied in epigenetic studies and is reviewed here.

**Fig. 1:**
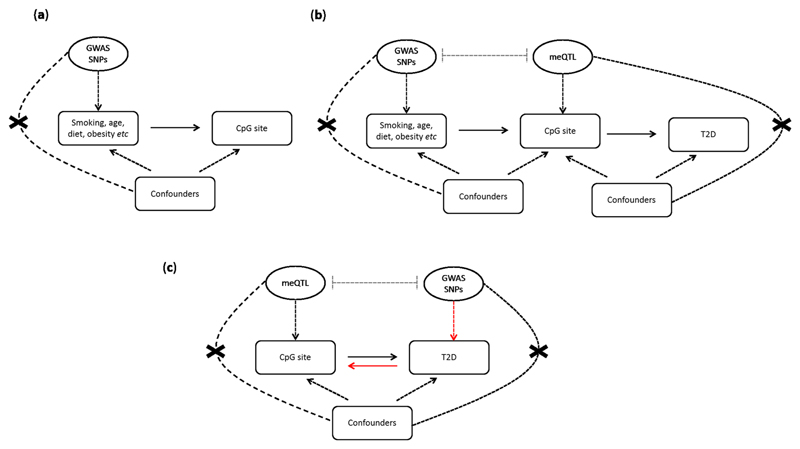
Example of how Mendelian randomisation can be applied to ascertain causality in epigenetic studies of T2D. **a** Investigate the causal role of known risk factors for T2D on variation in DNA methylation using EWAS evidence. Genetic proxies for the risk factor are extracted from the largest GWAS meta-analyses. These genetic variants should be independent of known confounders of the main association. **b** Use of MR to interrogate the mediating role of DNA methylation variation in the association between established risk factors and T2D. This design is known as a two-step epigenetic MR. The first step of the analysis calculates the causal effect of a risk factor on variation in DNA methylation based on EWAS findings and using GWAS loci to proxy variation in the exposure. The second step calculates the causal effect of DNA methylation (mediator) on T2D using independent methylation quantitative trait loci (meQTL acting in cis or trans) to proxy for variation in DNA methylation. meQTL are extracted from large studies of meQTL catalogues. Lastly, the mediated effect is calculated by multiplying the intermediate causal effects between the risk factor and DNA methylation, and between DNA methylation and T2D. **c** Applying bidirectional MR to investigate the causal direction of an observational association identified between DNA methylation and T2D in an EWAS

MR uses germline genetic variations as instrumental variables to establish the causal relationship between a modifiable exposure (in this case, DNAm) and a related outcome (in this case, T2D) in observational epidemiology [9, 80–82]. Because genetic variants are randomly transmitted from parents to offspring, they are fixed at conception and not influenced by behavioural, socioeconomic, or physiological factors commonly affecting observational associations, or by the disease itself through reverse causation [9, 80, 81]. MR can be applied to epigenetic studies in a number of different ways (Fig. 1); (a) to seek causal evidence of an exposure (e.g. smoking, alcohol intake, obesity) on methylation variation [78]; (b) to seek causal evidence of a mediating role of methylation variation on a disease outcome (e.g. smoking, methylation change, lung cancer) [83]; or (c) to asses directionality of an observed association when reverse cause is suspected [32•].

Sources of genetic instruments to conduct MR studies are GWAS of relevant exposures or traits, and studies identifying methylation quantitative trait loci (meQTL), which detect common genetic variants (SNPs) associated with variation in DNAm at CpG sites [9, 11, 84]. Due to the nature of DNAm, meQTL need to be identified in a temporal and tissue-specific manner, ideally consistent with the time-point and tissue where the epigenetic association was observed [9, 11, 84]. Large-scale meQTL studies have been conducted by Gaunt et al. (www.mqtldb.org) [84], Bonder et al. (BIOS QTL browser, https://genenetwork.nl/biosqtlbrowser/) [85], and most recently, by the genetics of DNAm consortium (GoDMC, www.godmc.org.uk/). Collectively, these studies provide a catalogue of known meQTL. However, they have the limitation that meQTL are exclusively derived from peripheral blood DNA. To date, the two largest consortia for the study of the genetics of T2D and glycemic traits are the Diabetes Genetics Replication and Meta-analysis (DIAGRAM, www.diagram-consortium.org) and the Meta-analysis of Glucose and Insulin-related traits consortium (MAGIC, www.magicinvestigators.org/).

Special considerations for the design of MR studies have been described elsewhere [80–82]. Based on the source of data used to derive effect estimates of the association between the genotype, the modifiable exposure, and the outcome, the MR approach can be a single sample MR (estimates from a single sample with individual-level data) or a two sample MR (estimates from two independent samples with summary data) [81, 82]. Previously, MR studies in T2D have been performed to understand the causal role of adiposity, blood lipids, and inflammatory risk factors on the disease [86]. However, as outlined above, MR can also be extended to study the causal role of DNAm as a mediator in the exposure-outcome association, or as the exposure or outcome of interest [77]. In either case, causality needs to be supported by identifying SNPs in strong association with methylation at the CpG site(s) of interest [11, 47•, 77, 78, 87].

Despite continues efforts to increase sample size, the power of current meQTL studies only allows identification of a small number of independent SNPs strongly associated with DNA methylation levels at CpG sites of interest [88]. This phenomenon imposes some limitations when using meQTL as instruments in MR studies due to the small variance in methylation captured by the meQTL (i.e. weak instrument bias), and the inability to conduct further sensitivity analyses to rule out confounding by horizontal pleiotropy [88]. Evidence to date supports a highly polygenic architecture of DNAm. Future datasets of meQTL are expected to provide stronger instruments for a larger number of CpG sites and will need to include the development of methods to allow the use of multiple meQTL in a single instrument whilst accounting for their likely correlation with each other. Approaches such as multiple trait colocalisation have proven useful in strengthening causal inference [88] but further methodological development is warranted. The risk of false positives can be reduced by conducting an indepth inspection of the associations identified drawing upon various sources of tissue-specific reference data for example.

In comparison with GWAS of complex traits that include large sample sizes, studies with availability of genetic and DNAm data are generally modest in size [47•]. In principle, having a small sample size can limit the use of DNAm in a single sample MR analysis, but this can be circumvented in a two sample MR design, where associations are retrieved from summary data using two independent and well-powered samples [47•].

#### Interaction Between Genetic and Epigenetic Variation in T2D

The role of the epigenome in regulating gene function is not independent of the genotype, as SNPs can influence methylation variance at CpG sites that also have a component of environmental variance [77]. In some instances, SNPs can affect methylation directly by introducing or removing a CpG site in the context of CpG-SNPs [23•, 89, 90], which have been identified in blood [84] and in T2D relevant target tissues [90–92]. Despite identifying SNP-DNAm associations at candidate loci for T2D [46, 47•, 90] and obesity [93], it is still unclear whether genetic variants affect both traits, DNAm and the disease, simultaneously or independently. In a study conducted by Elliott et al. [46], the principles of MR were used to ascertain the role of DNAm as a mediator in the association between the genotype (i.e. established GWAS SNPs for T2D) and future liability to T2D, based on methylation profiled in unaffected young participants [46]. Multiple CpG sites associated with T2D-SNPs were identified as potential non-causal biomarkers for T2D [46]. However, only for one site (mapping to the *KCNQ1* gene) was there any evidence that DNAm was on the causal pathway to disease in later life [46].

Instead of using T2D-SNPs as causal anchors to identify CpG sites associated with liability to T2D, Richardson et al. used meQTL as instrumental variables to ascertain the causal role of DNAm as a mediator in the genotype-T2D and genotype-glycemic traits association [47•]. meQTL were extracted from the mQTL database [84], while associations with the outcome were extracted from the latest GWAS meta-analysis in T2D [94] and glycemic traits [95, 96]. Analyses were performed using a two sample MR, and after multiple testing correction, a causal role of DNAm on T2D (at *p* < 1.39 × 10^−8^) was identified at CpG sites in cg04198914 (*HNF1B*), cg03864215 (*KCNJ11*), cg23956648 (*IGF2BP2*), and cg25064352 (*WFS1*) [47•], as well as at cg15453836 (*PEAK1*) and cg01883759 (*JAZF1*) [47•]. With respect to the glycemic traits, a causal effect of methylation on fasting proinsulin was detected at five CpG sites (in *PDE2A, PTPMT1, STARD10*, and *ARAP1*), and with HbA1c at seven CpG sites (in *G6PC2, TBCD*, and *FN3K*) [47•]. The use of colocalisation methods further indicated that the same causal variant was explaining variation in DNAm and T2D at *KCNJ11* and *WFS1*, while for the remaining loci, associations were explained by two different but correlated instruments [47•]. To ascertain the true direction of effect, a reverse MR (T2D➔DNAm) was conducted for associations with previous evidence of colocalization [94]. In this analysis, 25 SNPs identified in a recent GWAS meta-analysis for T2D were used as genetic instruments. Compared with results of the forward MR (DNAm➔ T2D), results of the reverse MR showed weaker evidence (*p* > 1.39× 10^−8^) that T2D was causally determining changes in DNAm at *KCNJ11* and *WFS1* [47•]. Overall, the study by Richardson et al. illustrates how MR methods can be used to prioritise DNAm markers with potential influence on T2D and related traits. However, CpG sites identified in this causal analysis cannot be regarded as true mediators of the SNP-T2D and SNP-glycemic trait associations, as possible horizontal pleiotropic effects (i.e. SNP-T2D association independent of DNAm) could not be completely ruled out, even after incorporating colocalisation methods.

#### Causal Effect of DNA Methylation on T2D and Related Traits Based on EWAS Findings

MR can also be applied to associations detected observationally using EWAS, e.g. for BMI [32•, 78, 87], although this approach has yet to be formally adopted for T2D. For BMI, MR analyses have demonstrated that changes in methylation are more likely to be a consequence of BMI rather than the cause [32•, 87].

A logical extension of this causal evidence (that the disease state impacts methylation and not vice versa) is that methylation variable loci may be informative in prediction of future comorbidities. In a study by Wahl et al. [32•], a methylation risk score generated using 11 CpG sites prospectively associated with BMI was able to predict future T2D risk (relative risk = 2.3, 95% CI = 2.07–2.56 per 1SD increase in the score) [32•].

Considering the growing evidence of methylation variable loci associated with T2D based on well-powered EWAS and meta-analyses of EWAS of T2D, it is necessary to strengthen evidence of the causal role of these signals using triangulation of causal inference methods, including MR, to prioritise candidate methylation loci for the early detection, adequate subtyping, and treatment of T2D. Even if they are not causal, CpG sites detected prospectively in association with T2D can be used as biomarkers based on the replicability of these associations across studies, and on their relevance in revealing new biological mechanisms of disease.

## Conclusion

Epigenetic studies of T2D offer a new avenue to discover novel biological mechanisms implicated in T2D aetiology alongside biomarkers of disease that are potentially informative for disease prediction. A number of loci have been detected in large-scale studies measured predominantly in blood, including *TXNIP, ABCG1, CPT1A*, and *SREBF1*. Methods to establish causality of epigenetic markers in T2D aetiology are becoming common-place. Because observational studies do not allow differentiation between causal and consequential epigenetic variation, triangulation of methods for assessing causality is increasingly informative. MR is a frequently used method for assessing causality that we have reviewed here. Ultimately, understanding the causality of epigenetic markers in T2D aids prioritisation of CpG sites as earlier biomarkers to detect disease, or in drug development to target epigenetic mechanisms in order to treat patients.

## Figures and Tables

**Table 1:** Characteristics of candidate gene studies and genome-wide studies associated with type 2 diabetes.

	Population	Study	Tissue	Method	Sample size	Gender	Replication CpG/DMR	Causal analysis	Results
***Candidate gene studies***									
Zou et al (2013) [40]	Chinese	Cross-sectional case control	Peripheral blood	MeDIP-Chip array	cases=152, controls=120	F/M	No	No	Hypermethylation of seven CpG sites in *PRKCZ* promoter and decreased expression of serum *PRKCZ* transcripts
Gu et al (2013) [41]	Swedish	Cross-sectional case control	Peripheral blood	Bisulphite pyrosequencing	cases=164, controls=242	M	No	No	Hypermethylation of 6 CpG sites in *IGFBP-1* in patients with overt T2D and newly diagnosed participants, and decreased serum expression of *IGFBP-1*. Higher methylation in newly diagnosed participants with a FH of T2D compared to those without FH of T2D. No difference in methylation relative to the treatment used in T2D-treated participants.
Gu et al (2013) [42]	Swedish	Cross-sectional case control	Peripheral blood	Bisulphite pyrosequencing	cases=240, controls=100	F/M	No	No	Hypermethylation of *IGFBP-7* in T2D cases (men) with or without treatment for glucose control. No correlation between DNAm and gene expression at *IGFBP-7*.
Canivell et al (2014) [43]	Spanish	Cross-sectional case control	Peripheral blood	EpiTYPER assay	cases=93, controls=93	F/M	No	No	Differential methylation at 13 CpG sites in the promoter of *TCF7L2* between treatment naïve cases and age- and BMI-matched controls. Correlation between methylation at specific sites and fasting glucose, HOMA scores, LDL-chol and total cholesterol.
Gemma et al (2010) [39]	Argentinian (European descent)	Cross-sectional case control	Peripheral blood	Bisulphite pyrosequencing and MS-PCR	IR=77, NIR=45	F/M	No	No	Hypomethylation in the promoter of *TFAM* (3 sites), the mitochondrial transcription factor A, was inversely associated with insulin resistance in young adults. Inverse correlation between methylation of *TFAM* and different measures of insulin resistance (fasting insulin, HOMA-IR and obesity).
Seman et al (2015) [45]	Malaysian	Cross-sectional case control	Peripheral blood	Bisulphite pyrosequencing	Cases=509, controls=441	F/M	No	No	Hypermethylation of 5 CpG sites in the promoter of *SLC30A8*. Combined effect of 6 sites in a methylation score was higher in T2D cases compared to controls.
Yang et al (2011) [35]	Swedish	Cross-sectional case control		Bisulphite treatment,	Cases=9, controls=48	F/M	No	No	Hypermethylation of 4/25 sites near the TSS in the INS promoter. Inverse correlation between methylation at three CpG sites in INS and INS expression, while positive correlation between INS methylation and HbA1c levels.
			Human pancreatic islets	EpiTYPER assay					
Yang et al (2012) [36]	Swedish	Cross-sectional case control	Human pancreatic islets	Bisulphite treatment, EpiTYPER assay, Pyrosequencing (CpG's in promoter)	Cases=9, controls=55	F/M	No	No	Hypermethylation of 10 CpG sites in the promoter and enhancer regions of PDX1 in T2D cases. Inverse correlation between methylation of PDX1 and PDX1 expression. Positive correlation between PDX1 methylation and HbA1c levels. Functional analysis using clonal human β- and α-cells, further confirmed the associations between DNAm, mRNA expression and hyperglycemia.
Ling et al (2008) [34]	Swedish	Cross-sectional case control	Human pancreatic islets	Bisulphite treatment, PCR amplification, cloning and sequencing of amplicons	Cases=10, controls=9	F/M	No	No	Hypermethylation of 4 sites in the promoter of PPARGC1A in association with T2D. In addition, decreased PPARGC1A expression and insulin secretion in T2D donors.
Hall et al (2013) [37]	Swedish	Cross-sectional case control	Human pancreatic islets	Bisulphite treatment, EpiTYPER assay	Cases=10, controls=55	F/M	No	No	Hypermethylation of 1 site (out of 18 analyzed) near the TSS of GLP1R in T2D donors, and correlation of methylation at a second CpG site in GLP1R with GLP1R expression (-), HbA1c levels (+) and BMI (+)
Kulkarni et al (2012) [38]	Swedish	Cross-sectional case control	Skeletal muscle biopsies	Bisulphite treatment, PCR amplification, cloning, and sequencing of amplicons	Cases=33, controls=79	F/M	No	No	Hypomethylation in the promoter of PDK4 in T2D donors, and increased PDK4 expression. Positive correlation between PDK4 expression and BMI, fasting and 2-h glucose, fasting insulin, C peptide and HbA1c across groups.
**Epigenome-wide association studies: incident T2D**									
Chambers et al (2015) [16•]	a. Indian-Asians b. Europeans (LOLIPOP)	Longitudinal nested case control study	Peripheral blood	450K	Discovery: incident cases=1608, controls=11927; Replication: incident cases=306, controls=6760	F/M	Yes (5 CpG sites)	No	7 sites identified with genome-wide significance (p<5.0x10^-7^) in the discovery study. Replication of 5/7 sites (p<1.0x10^-7^), where risk of future T2D was increased per 1% increase in methylation at *ABCG1* (cg06500161) [RR=1.09, 95%CI=1.07-1.11] and *SREBF1* (cg11024682) [RR=1.07, 95%CI=1.04-1.09], while future risk of T2D decreased per 1% increase in methylation at *TXNIP* (cg19693031) [RR=0.92, 95%CI=0.90-0.94], *PHOSPHO1* (cg02650017) [RR=0.94, 95%CI=0.92-0.95] and *SOCS3* (cg18181703) [RR=0.94, 95%CI=0.92-0.96]. *TXNIP* association surpassed further adjustment for non-genetic established risk factors for T2D. Cross-tissue correlation in the levels of methylation for *TXNIP* and *SOCS3* (blood vs liver). DNAm-gene expression association in blood: *ABCG1* and *SREBF1*, and in liver: *TXNIP*.
Dayeh et al (2016) [54]	Finnish	Longitudinal nested case control study	Discovery: Peripheral blood; Validation: T2D relevant tissues	Pyrosequencing, 450K	Incident cases=129, controls=129	F/M	No. Designed to replicate signals in Chambers et al. [11]	No	Directional replication of associations at *ABCG1* [OR=1.09, 95%CI=1.02-1.16] and *PHOSPHO1* [OR=0.85, 95%CI=0.75-0.95] for incident T2D based on previous findings. Methylation of *ABCG1* was positively correlated with BMI, HbA1c, triglycerides and fasting insulin, while *PHOSPHO1* was positively correlated with HDL. Inverse correlation between gene expression and methylation in muscle for *ABCG1*. A methylation score combining the effect of 5 sites initially investigated could not predict future risk of T2D (p=0.22).
Jeon et al (2017) [58]	Korean	Cross-sectional case control	Peripheral blood (discovery) and pancreatic islets (replication)	a.450K b.Pyrosequencing	Discovery: High-glucose group=8, T2D=5, controls=13; Replication: T2D=220, controls=220	F/M	Yes (1 CpG site)	No	Hypomethylation of *MSI2* and *CXXC4* in T2D and high-glucose group compared to controls. Replication of *MSI2* association with T2D in blood samples from an independent cohort. *MSI2* hypomethylation was also seen in pancreatic islets of T2D donors, indicating its relevance in T2D. Knockdown/overexpression of *MSI2* in mouse β-cells, resulted in altered insulin expression, meaning that *MSI2* could have a regulatory role in T2D.
**Epigenome-wide association studies: prevalent T2D**									
Toperoff et al (2012) [48]	Ashkenazi Jews	Cross-sectional case control study (discovery); longitudinal study (replication)	Peripheral blood	Microarray-based assay, Sequencing of bisulphite converted DNA pools	Discovery: cases=710, controls=459; Replication: incident IGM=58, controls=64	F/M	Yes (13 CpG sites)	No	Six DMRs differentially methylated in association with T2D. Replication of significant DMRs identifying 13/93 CpG sites with strong differences between T2D cases and controls at *THADA*, *JAZF1*, *FTO*, *SLC30A8*, *TCF7L2* and *KCNQ1* genes. The strongest replicated signal was at a CpG in the first intron of FTO, where hypomethylation was associated with 6.1% higher risk of T2D. Replicated associations were confirmed in a prospective study of young participants who later developed T2D.
Yuan et al (2014) [52]	European (UK)	Cross-sectional case control (twin study)	Peripheral blood	a.MeDIP-seq, b.450K	Discovery: 27 twin pairs (17 T2D-discordant MZ twins, 3 T2D-concordant MZ twins and 7 control pairs); Replication: cases=42, controls=221	F/M	Yes (1 DMR)	No	DMRs in association with prevalent T2D were mostly hypermethylated and located in candidate loci: *THADA*, *FTO*, *IRS1*, *ADAMTS9*, *SLC30A8* and *KCNJ11*. The strongest replicated DMR in unrelated participants mapped to the *MALT1* gene, and an additional DMR was identified in the GPR6 gene.
Kulkarni et al (2015) [15]	Mexican Americans	Cross-sectional family-based case control	Peripheral blood	450K	cases=174, controls=676	F/M	No	No	Identification of 51 CpG sites associated with T2D showing minimal variation in methylation (top sites at *ABCG1*, *SAMD12*, *TXNIP* and two intergenic CpG’s, median difference in methylation=2-5%); 19 associated with fasting glucose and 24 with HOMA-IR. Validation of *TXNIP* and *ABCG1* associations using pyrosequencing. *TXNIP* was also associated with fasting glucose and HOMA-IR, while *ABCG1* was associated with HOMA-IR after adjustment for covariates.
Soriano-Tarraga et al (2016) [55]	Spanish	Cross-sectional case control	Peripheral blood	450K	Discovery: cases=151, controls=204; Replication (BISMAR_2): cases=59, controls=108; Replication (REGICOR): cases=63, controls=582	F/M	Yes (1 CpG site)	No	Association of *TXNIP*, *POR* (cg01676795), *PFKFB3* (cg26262157), and the intergenic CpG site cg07805383, with prevalent T2D in discovery cohort. Replication of *TXNIP* in two independent cohorts. *TXNIP* inversely associated with HbA1c levels in T2D patients with poor control of glucose levels.
Florath et al (2016) [29•]	German	Cross-sectional case control	Peripheral blood	450K	Discovery: cases=153, controls=835; Replication: cases=87, controls=527	F/M	Yes (1 CpG site)	No	Association between T2D and 39 CpG sites in discovery cohort (top 5 sites at *RNU5E*, *NFE2L3*, cg07133434, cg17156491 and cg23919742). Replication of *TXNIP* association in a sub-cohort from the ESTHER study. *TXNIP* was inversely associated with fasting glucose and HbA1c in a dose-response manner. *TXNIP* methylation was 5% lower in T2D patients with poor control of glucose (HbA1c>7.0%), compared to those with adequate glucose levels.
Al Muftah et al (2016) [49]	a.Arab, b.Caucasian	Cross-sectional family-based case control	Peripheral blood	450K	Discovery: cases=30, controls=93; Replication: 810 T2D-discordant MZ twin-pair females	Discovery: F/M; Replication: F	Yes (2 CpG site)	No	Novel T2D loci identified at *DQX1* (cg06721411) [p=1.18x10-9], which was replicated in a subsample of the TwinsUK study. Additionally, replication of the well-known signal at *TXNIP* in the Qatari study and the TwinsUK study [meta-analysis beta=-0.43, p=2.71x10^-4^].
Walaszczyk et al (2018) [27•]	Dutch	Cross-sectional case-control	Peripheral blood	450K	Cases=100, controls=100. Sensitivity analyses: cases=50, controls=50	F/M	No. Designed to confirm sites from a literature review [23].	No	Replication of CpG sites in *ABCG1*, *LOXL2*, *TXNIP*, *SLC1A5* and *SREBF1* in association with T2D, and CpG sites in *ABCG1* and *CCDC57* in association with fasting glucose, using a subsample from the LIFELINES study. BMI was identified as a confounder in most of the replicated associations, and none of the tissue-specific CpG sites were replicated in peripheral blood. Correlation was seen between T2D-replicated CpG sites and glycemic and lipid phenotypes in healthy participants. Overlap between T2D-sites, and sites detected in EWAS of BMI and lipids.
Meeks et al (2018) [56•]	African (Ghana)	Cross-sectional case-control	Peripheral blood	450K	Cases=256, controls=457	F/M	No	No	After adjustment for common covariates and correction for inflation and multiple testing, significant associations were identified at four loci: *TXNIP* (cg19693031), *C7orf50* (cg04816311), *CPT1A* (cg00574958) and TPM4 (cg07988171), which together explained 25% of the variance in T2D. The strongest association with T2D was reported at the *TXNIP* locus, and further adjustment for BMI resulted in three significant associations: *TXNIP*, *TPM4* and *C7orf50*. A DMR was identified at the *GDF7* gene [n=7 sites, OR=4.37, 95%CI=1.43-13.38], which explained 1.2% of the variance in T2D.
Nilsson et al (2014) [61]	European	Cross-sectional case-control	Adipose tissue	450K	Discovery: 14 T2D-discordant MZ twins; Replication: cases=28, controls=28	F/M	No	No	Detection of modest differences in methylation in adipose tissue at 23470 sites, none of them surpassing FDR correction. Analysis in unrelated participants resulted in the detection of DNAm differences with FDR significance in 7046 genes, some of them matching with GWAS loci for T2D (*PPARG, KCNQ1, TCF7L2*, and *IRS1*). Methylation was associated with expression of the same gene for 266 loci.
Dayeh et al (2014) [65]	European	Cross-sectional case-control	Human pancreatic islets	450K	Cases15, controls=34	F/M	No	No	Detection of over 3 000 CpG sites with differential methylation in association with T2D in human islets. Average difference in methylation was >5%. CpG sites were identified in known GWAS loci for T2D (*ADAMTS9, ADCY5, FTO, HHEX, HNF1B, IRS1, JAZF1, KCNQ1*, etc.), and in novel loci (*CDKN1A, PDE7B, EXOC3L2* and *HDAC7*). DNAm was associated with differential gene expression in some loci like *HDAC7* (hypomethylated and overexpressed).
Volkov et al (2017) [67]	European	Cross-sectional case-control	Human pancreatic islets	WGBS	Cases=6, controls=8	F/M	No	No	~ 26 000 DMRs were identified in association with T2D, the average size for a DMR was 414 bp (6 bp-3411 bp), and they were in genes related with β-cell function (*PDX1, TCF7L2* and *ADCY5*), and within known GWAS loci for T2D. DMRs were also enriched in binding sites for transcription factors, indicating their role in regulating islet function. For some of the DMRs, changes in methylation were reflected on changes in gene expression in T2D islets (i.e. DMRs in *NR4A3, PARK2, PID1*, and *SOCS2*). Highest difference in methylation (>10%) between T2D cases and controls in DMRs annotated to *ARX* and *TFAM* genes.
Ribel-Madsen et al (2012) [59]	Danish	Cross-sectional case control (twin study)	Skeletal muscle	27K	11 T2D-discordant MZ twin pairs	F/M	No	No	Differences in methylation identified in the *IL8* and *PPARGC1A* genes.
Nilsson et al (2015) [70]	European (Finland)	Cross-sectional case-control	Liver	450K	Cases= 35, controls=60	F/M	No	No	251 CpG sites differentially methylated in T2D cases compared to controls (Q-val<0.05), most of them hypomethylated in T2D donors. Hypomethylation in T2D was potentially due to reduced levels of erythrocyte folate, a dietary donor of methyl groups, in T2D donors. Some CpG sites mapping to known GWAS loci in T2D (i.e. *GRB10, ABCC3, MOGAT1* and *PRDM16*). Two of the genes with differential methylation and expression in the liver were *H19* and *RIPK4*. *RIPK4* has been previously related with decreased insulin sensitivity in the liver.

a. Discovery cohort/method, b. Replication study/validation method. F, females; M, males; T2D, type 2 diabetes; MZ, monozygotic twins; MeDIP-seq, methylated DNA immunoprecipitation sequencing; MS-PCR, methylation-specific polymerase chain reaction; WGBS, whole genome bisulphite sequencing; IR, insulin resistance; NIR, noninsulin resistance; TSS, transcription start site; HbA1c, hemoglobin A1c; IGM, impaired glucose metabolism based on fasting plasma glucose levels ≥ 110 mg/dl and/or ≥ 140 mg/dl 2h after a 75g oral glucose load; LOLIPOP, London life sciences prospective population study; FHS, Framingham heart study; RR, relative risk; BD-MR, bidirectional Mendelian randomization; PRKCZ, protein kinase C zeta; IGFBP-1, insulin-like growth factor binding protein-1; IGFBP-7, insulin-like growth factor binding protein-7; TCF7L2, transcription factor 7-like 2 protein; TFAM, mitochondrial transcription factor A gene; PPARGC1A, the peroxisome proliferator activated receptor gamma coactivator-1 alpha; GLP1R, glucagon-like peptide-1 receptor; INS, insulin gene; PDX1, pancreatic duodenal homeobox 1; PDK4, pyruvate dehydrogenase kinase 4; MALT1, mucosa-associated lymphoid tissue lymphoma translocation protein 1; GPR6, G-protein receptor 6 gene; TXNIP, thioredoxin interacting protein; ABCG1, ATP-binding cassette sub-family G member 1; PHOSPHO1, phosphoethanolamine/phosphocholine phosphatase 1; SOCS3, suppressor of cytokine signaling 3; SREBF1, sterol regulatory element-binding transcription factor 1; SAMD12, Sterile alpha motif domain containing 12; POR, Cytochrome P450 Oxidoreductase; PFKFB3, 6-Phosphofructo-2-Kinase/Fructose-2,6-Biphosphatase 3; TPM4, Tropomyosin 4; MSI2, Musashi RNA Binding Protein 2; CXXC4, CXXC finger protein 4; ARX, Aristaless Related Homeobox; TFAM, Transcription Factor A, Mitochondrial; IL8, C-X-C motif chemokine ligand 8.
